# Nonparametric Evaluation of Dynamic Disease Risk: A Spatio-Temporal Kernel Approach

**DOI:** 10.1371/journal.pone.0017381

**Published:** 2011-03-15

**Authors:** Zhijie Zhang, Dongmei Chen, Wenbao Liu, Jeffrey S. Racine, SengHuat Ong, Yue Chen, Genming Zhao, Qingwu Jiang

**Affiliations:** 1 Department of Geography, Queen's University, Kingston, Ontario, Canada; 2 Department of Epidemiology, Fudan University, Shanghai, People's Republic of China; 3 Department of Economics, McMaster University, Hamilton, Ontario, Canada; 4 Department of Mathematical Sciences, University of Malaya, Kuala Lumpur, Malaysia; 5 Department of Epidemiology and Community Medicine, University of Ottawa, Ottawa, Ontario, Canada; University of KwaZulu-Natal, South Africa

## Abstract

Quantifying the distributions of disease risk in space and time jointly is a key element for understanding spatio-temporal phenomena while also having the potential to enhance our understanding of epidemiologic trajectories. However, most studies to date have neglected time dimension and focus instead on the “average” spatial pattern of disease risk, thereby masking time trajectories of disease risk. In this study we propose a new idea titled “spatio-temporal kernel density estimation (stKDE)” that employs hybrid kernel (i.e., weight) functions to evaluate the spatio-temporal disease risks. This approach not only can make full use of sample data but also “borrows” information in a particular manner from neighboring points both in space and time via appropriate choice of kernel functions. Monte Carlo simulations show that the proposed method performs substantially better than the traditional (i.e., frequency-based) kernel density estimation (trKDE) which has been used in applied settings while two illustrative examples demonstrate that the proposed approach can yield superior results compared to the popular trKDE approach. In addition, there exist various possibilities for improving and extending this method.

## Introduction

Modern epidemiology is founded on spatial analysis that can be traced back to the classical paradigm of John Snow's work on cholera in the middle of nineteenth century [1]. However, only in the past two decades have advances in geographic information systems (GIS) and in statistical methods for analyzing spatially-referenced health data allowed epidemiologists to re-evaluate spatial epidemiology from the perspective of visualizing the trajectory of disease risk across space and time [2]. Among others, the kernel density estimation (KDE) based spatial relative risk function (sRRF) have attracted much attention because of its flexibility in applications and its minimal assumptions regarding the underlying data structure [3]. In 1990, Bithell first introduced the method of kernel density ratio between cases and controls into the field of epidemiology for describing the spatial relative risks [4], [5]. Kelsall and Diggle further developed the 1-dimensional case [6] and also extended it to the 2-dimensional spatial settings [7]. More recently, the ratio of adaptive kernel density estimation has been proposed to depict the spatial variation of disease risk [8], [9]. In addition to the theoretical development, there have been many successful applications of sRRF in human and veterinary epidemiology. For example, Sabel *et al.* studied the spatial pattern of motor neurone disease risk in Finland [10], Prince *et al.* examined the geographic risk of primary biliary cirrhosis in a region of north-east England [11], Wheeler detected the childhood leukemia clustering and clusters in the US state of Ohio [12], Berke generated the relative risk maps of pseudorabies-seropositive (Aujeszky's disease) pig herds in an animal-dense region of Germany [13], while Zhang *et al.* assessed the schistosomiasis risk in a region of Anhui province in China [3]. Nowadays, spatial epidemiology is increasingly being used to assess disease risk, but the patterns of disease risk tend to have both spatial and temporal components [14]. The risk pattern in discrete time dimension has always been neglected [2].

In recent years, there has been a growing interest in integrating temporal information in GIS, while advances in computing technologies have made it possible to implement many of the new concepts developed to address temporal problems [15]. This has led to the increasing availability of spatio-temporal disease data sets where the risk patterns in space and time need to be considered simultaneously [16], [17]. The above mentioned KDE-based idea is well-suited for such problem. It is obvious that spatio-temporal data are mixed variable types comprised of continuous spatial variables and an ordered categorical time variable. Unfortunately, the conventional KDE presumes that the underlying variable is continuous in nature, and a widely used approach in such settings is the traditional “frequency” based KDE (trKDE) [7], [9], [10]. trKDE first splits the samples into subsets, one for each realization of the time variable so that each subset contains only the continuous spatial variables, and then traditional kernel estimation is conducted for each subset. Though statistically consistent, one may often encounter sparse subsets containing insufficient data to deliver accurate nonparametric density estimates [18].

This paper introduces spatio-temporal kernel density estimation (stKDE) based on the concept of “generalized product kernels”. Ttraditional product kernels only allows the variables with the same data types, while this “generalized product kernels” relax this constraint by allowing different data types. Rather than breaking related data into subsets and modeling the density of continuous variable only, this approach models the full (i.e. joint) spatio-temporal density and smooth the ordered time variable in an appropriate way. Monte Carlo simulations and two epidemiological examples were first provided to assess the method that is used to determining the spatio-temporal variation in disease risks.

## Materials and Methods

### 2.1 Spatio-temporal kernel density estimation (stKDE)

Let 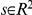 represents the continuous variables of spatial coordinates, and 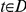 denotes time as an ordered discrete variable. Let 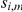 be the *m*th component of 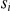 and let 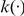 be a univariate kernel function for continuous data (e.g. Epanechnikov), the traditional product kernel 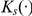 for the continuous spatial variables is [19],
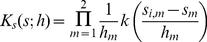
(1)Where *h*_m_ represent the smoothing parameters or bandwidths for spatial coordinates x/y; *i* = 1,2,…*n* mean the corresponding study participants (cases or controls) and *m* = 1,2 are the spatial variables of x/y coordinates.

Let 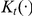 indicate the univariate kernel of the ordered time variable *t*. Wang and van Ryzin's method of constructing the kernel to reflect the ordinals of variable was adopted here and is given by [20],
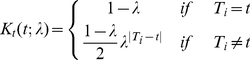
(2)Where 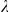 represents the smoothing parameter of this ordered kernel.

Then the generalized spatio-temporal product kernel 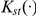 for two continuous and one ordered variable is defined as follows [21], [22],
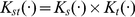
(3)Where 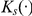 and 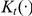 are from equations (1) and (2), respectively. More mathematical and theoretical details on the properties of generalized product kernels can be found in our previous reports [21]–[26].

Based on the above generalized product kernel, the method of trKDE can be logically extended to stKDE by treating the spatio-temporal data as mixed variable types using formula (4),
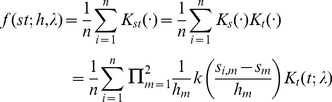
(4)Theoretical underpinnings for this estimator can be found in Text S1 or [21].

It is well known that for kernel-based estimators, selecting an appropriate bandwidth is a key element of sound estimation. In this study, the likelihood cross-validation (CV) was used to select the smoothing parameter *h*, which has been extensively recommended in the trKDE settings [27], [28]. Likelihood cross validation is a fully automatic and data-driven method and involves choosing those bandwidths that maximize the following formula [22]

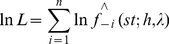
(5)Where 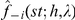 is the leave-one-out kernel estimator of stKDE in equation (4). It has been shown that this method of bandwidth selection is optimal in the Kullback-Leibler sense.

### 2.2 Spatio-temporal relative risk function (stRRF) and significance test

Next, suppose we have a dataset consist of two sets of points 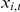:*i* = 1,2,…*n_1_* (cases), and 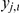:*j* = 1,2,…*n_2_* (controls) on a two dimensional region 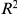 observed for several time periods (*t* = 1,2,…,l). Following Bithell's raw idea of a relative risk function (RRF) [5], the stRRF 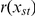 can be generated in equation (6) by simply taking the ratio of case and control stKDE in equation (4),
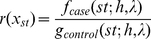
(6)To mitigate scaling problems that might arise in the presence of extreme values and to improve symmetry, the logarithms of 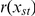 have been proposed [6], [7], and to reduce possible errors that might arise when the density approaches zero, a stabilization constant is also advocated [5] (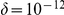 was used here). Hence the stRRF is defined as follows,
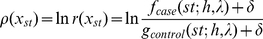
(7)Where 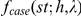 and 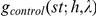 denote case and control stKDE, respectively, which are obtained by equation (4). 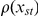 from equation (7) based on the collected cases and controls is actually an observed risk which we denote 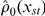. In order to assess the statistical significance of local peaks and troughs and also prevent over-interpretations of false positive results, we adopt the use of point-wise tolerance contours. In essence, under the null hypothesis of no spatial-temporal variations of disease risk, the cases and controls are drawn from a common distribution. There we randomly split the data into two groups of simulated cases *n*_1_ and controls *n*_2_, and then randomly reallocate the simulated cases and controls into each time cell based on its original sample size to thereby generate a simulated spatio-temporal data set under the null hypothesis of no spatio-temporal variation. Then, we can construct the simulated stRRF as before. Using this randomization mechanism we can repeat this process a number of times via Monte Carlo simulations. After performing this simulations *M* times, for each point we obtain a sorted series of simulated risk values (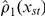, 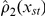, …, 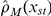) drawn under the null, then the *p* value for each position can be calculated using the formula, 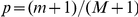 (8) (*m* is rank of 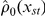 among all the simulated risk values) and *p*-value surface for the study area in each time can be obtained accordingly. The 2.5% and 97.5% contours from this *p*-value surface can be further extracted and overlaid on the map of 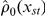 to highlight the regions with significantly low and high risks for a two-sided statistical test. For one-sided test, the 5% or 95% contour is used to detect the significance of low or high risks, respectively. This is the so-called approach of 95% point-wise tolerance contours. More details can be found in previous work [3], [6]–[8], [29].

### 2.3 Monte Carlo simulations

To investigate the performance of our proposed stKDE approach in finite-sample settings, four simulation studies were conducted. For each simulation, the samples were first randomly generated from the 3-dimensional multivariate normal distribution whose parameters were summarized in Table 1 and then one of the variables was converted into an ordinal categorical variable. This categorical variable and the other two untransformed continuous variables were used to simulate a spatio-temporal dataset with mixed variable types. Eight different sample sizes were used, 50,100,150,200,300,600,900 and 1200; three different levels of 2, 4 and 6 for the ordered variables were simulated. So there are 96 combinations in all, and 100 Monte Carlo replications were conducted for each combination.

**Table 1:** Distribution parameters of four simulations.

Simulation	Distribution	Mean vector	Covariance
1	Independent identical distribution	0,0,0	1,0,0/0,1,0/0,0,1
2	Independent shifted distribution	0,1,2	1,0,0/0,2,0/0,0,3
3	Dependent identical distribution	0,0,0	1,.5,.7/.5,1,.8/.7,.8,1
4	Dependent shifted distribution	0,1,2	1,.5,.7/.5,2,.8/.7,.8,3

Smoothing parameters were selected via likelihood cross validation using a conjugate gradient search algorithm to avoid local maxima and minima, and second-order Gaussian kernel for the continuous variables [30] and Wang and van Ryzin's kernel for ordinal variable were used to calculate the stKDE as defined above [20]. For the purpose of comparison, the trKDE was also calculated by dividing the samples into different subsets based on the levels of the ordered variable, and then trKDE was applied in each separate time level (subset). Mean integrated square error (MISE) was adopted to assess their performance and was computed via 
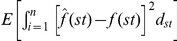
 for stKDE and 
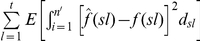
 for trKDE (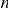 and 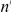 mean the total sample size and the individual sample size of each time level, respectively). Grouped box plots were generated to summarize the MISE of stKDE and trKDE, respectively.

### 2.4 Epidemiological examples

Two epidemiological examples were analyzed to demonstrate the value added by the proposed approach in applied settings through stKDE-based stRRF and 95% pointwise tolerance contours. One dataset is on Burkitt's lymphoma in Uganda and another considers schistosomiasis in China.

Burkitt's lymphoma is a common cancer in eastern Africa and has attracted epidemiological attentions for many years [31]. This dataset used in this study is comprised of 188 cases of Burkitt's lymphoma in the Western Nile district of Uganda for the period of 1961–1975. Accompanying the cases, the onset date of the disease and transformed spatial coordinates of numeric grid easting X and northing Y for confidential reasons were also available. So a spatio-temporal dataset with three variables of the onset year of disease (YEAR) and spatial locations (X and Y) could be built [32], [33]. But no controls were obtainable. To account for the population-at-risk distribution, the same sample size of controls as that of cases were randomly selected in that region which was assumed to represent the distribution of population at risk and the same controls were used in different years by assuming the at risk population were stable during the study period.

Schistosomiasis japonica is one of the most important parasitic diseases and has significant public health and socioeconomic impacts in China [34]. The schistosomiasis data set was from our previous studies in the Guichi region of Anhui province in China. All the acute schistosomiasis cases among permanent residents of that region from 2001–2006 were collected and the same sample size of controls were obtained by the probability proportion to size sampling approach that represent the underlying at-risk population distribution that gave rise to the cases [3], [35], [36]. All the spatial positions for cases and controls were obtained by the hand-held global positioning system (GPS) and the years the cases occurred were also retrieved from the raw records to establish the spatio-temporal dataset. The same controls were used in different years with an assumption that the at risk population were stable during the study period.

Both stKDE- and trKDE-based relative risk functions were used to analyze these two examples and 95% pointwise contour lines were added to the risk maps to highlight the statistically significant “peak” regions.

## Results

### 3.1 Simulation results on comparisons of stKDE and trKDE

**Figure 1:**
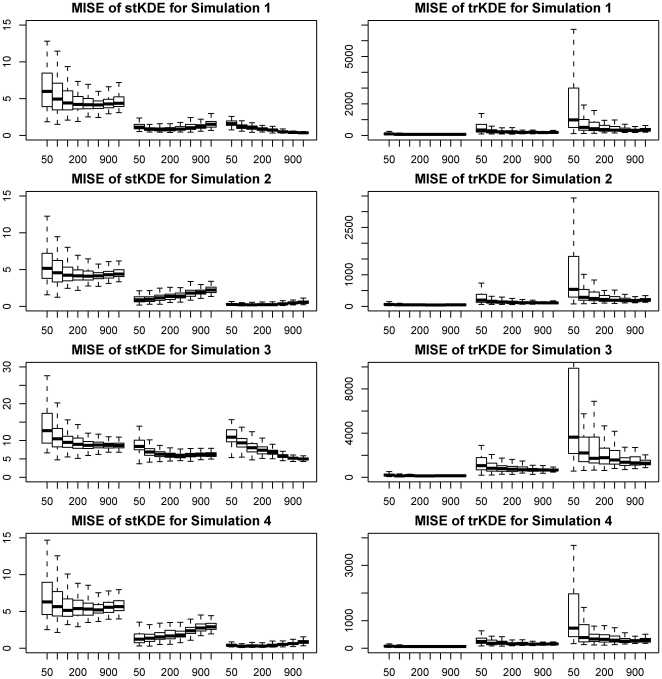
Grouped box plots of MISE for simulations of stKDE and trKDE. Box-whisker plots of MISE for different sample sizes and factor levels of ordered variable were grouped together for a clear comparison. The middle bold band inside the box is the 50^th^ percentiles or median; the bottom and top of the box are the 25^th^ and 75^th^ percentiles, that is, the lower and upper quartiles, respectively. The whiskers in the bottom and top are the values of 1.5 inter-quartile range (IQR) times the lower and upper quartiles. The y-axis represents the MISE and the x-axis is the sample sizes for three different levels of ordered time variable, which are 2, 4 and 6 levels in turn from left to right.

The simulation results are displayed with grouped box plots in Figure 1. The actual MISE values are available upon request. Figure 1 shows that stKDE consistently outperforms trKDE for all combinations of different levels of ordered variable and sample sizes given its smaller finite-sample MISE. And its performance improves as the number of levels for the ordered variable increases, that is, the ordered trait of the categorical variable becomes more pronounced. For the sample sizes considered, the MISE of trKDE tends to decrease for a given level of the ordered variable as the sample size increases as one would naturally expect (both approaches are consistent, stKDE is more efficient).

Furthermore, if we examine variation in MISE (say, by the inter-quartile range i.e. the length of the “box” in the boxplot), stKDE has an obvious narrower variation than trKDE that shrinks with the augmented number of levels of the ordered variable. Naturally, this variation decreases as the sample size increases for both stKDE and trKDE.

Besides, we observe that the results of simulation 1 and 3 (group1) are similar, while simulation 2 and 4 (group2) are similar for both stKDE and trKDE. And group 2 (shifted distributions) performs slightly better than that of group 1 (identical distributions). For individual MISE in groups 1 and 2, both stKDE and trKDE perform slightly worse for the dependent simulations 3 and 4 than independent simulations 1 and 2, respectively, as one would expect. This means the distribution properties and data dependence can influence the performance of stKDE and trKDE, but the overall influence on stKDE seems to be weaker than that on trKDE.

### 3.2 Epidemiological examples

**Figure 2:**
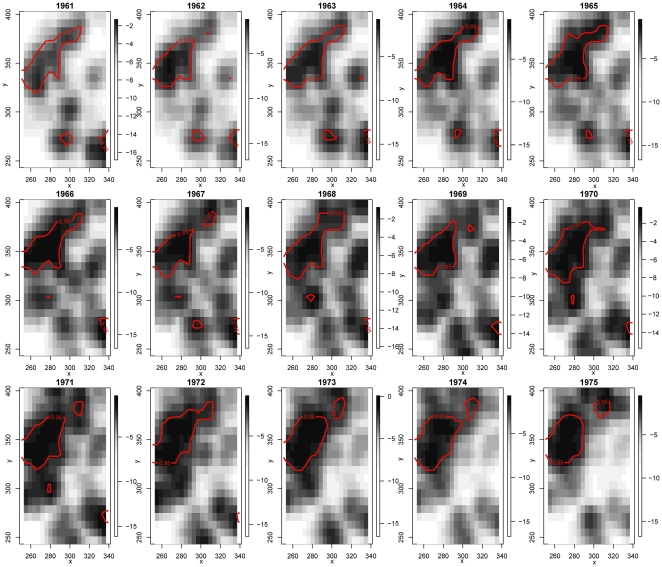
Spatio-temporal relative risk surface showing the risk changes of Burkitt's lymphoma in the Western Nile district of Uganda from 1961–1975. The degree of risk is denoted by the shade of gray with black shading representing the highest risk and the white the least risk. The solid contour lines delineate the significant high risk regions.

The numbers of Burkitt's lymphoma cases from 1961 to 1975 are 5, 4, 6, 12, 8, 20, 11, 12, 14, 15, 21, 15, 22, 5 and 18, respectively. Clearly, this is the case where data in each period are sparse, hence we would expect that modeling the joint distribution of the spatial and temporal variables would be immediately apparent. From the stKDE results displayed in Figure 2, we have the following three major observations. Firstly, there is a significantly high risk region in northwest of the study area which is stable among all the 15 years. Secondly, in the southern part and southeastern part, two significantly high risk regions can be seen intermittently, but disappeared after 1972. Finally, the overall risk in the study area seemed to increase gradually and reached a peak during 1966–1970 and then gradually decreases. While the results of trKDE are quite tenuous as expected, see Figure S1 for the plot.

**Figure 3:**
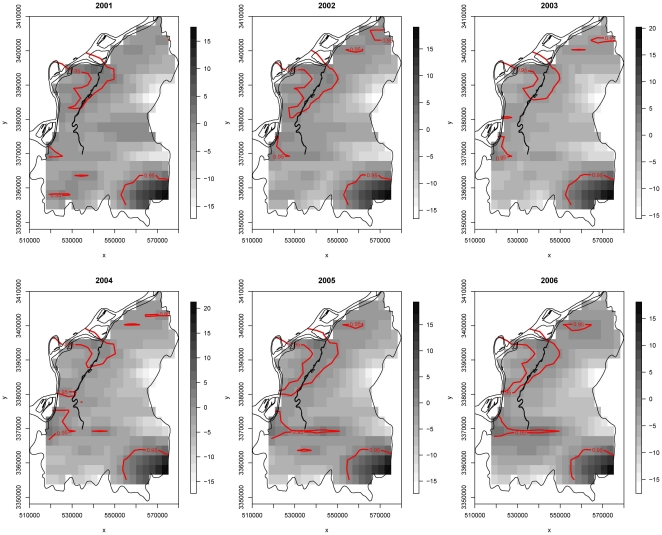
Spatio-temporal relative risk surface depicting the dynamic changes of schistosomiasis risk in the Guichi region of China from 2001–2006. The degree of risk is denoted by the shade of gray with black shading representing the highest risk and the white the least risk. The solid contour lines delineate the significant high risk regions.

The numbers of schistosomiasis cases from 2001 to 2006 are 13, 23, 13, 14, 14 and 6, respectively [36], and again the scarcity of data for each time period is obvious. From the stKDE results in Figure 3, four significantly high risk regions consistently appeared including two large regions in northeast and southwest, and two small regions in northeast and southwest. The former two regions are more stable in the study period of six years both in size and shapes, which may prompt that the risk factors in those regions are steady. The latter two regions are more variable, suggesting that the risk factors in these regions are dynamic. Hence, different control strategy might be warranted. Similar to the Burkitt's lymphoma example, the results of trKDE for schistosomiasis are also quite tenuous; see Figure S2 for the resulting plot.

## Discussion

Spatial epidemiology can be used to investigate the spatial distribution of diseases for detecting and highlighting areas with elevated disease risk, and to examine ecological risk factors for disease transmission. This is helpful in making rational disease control strategies and for effective allocation of resources [3], [37]. Most studies consider data aggregated over a period of time and therefore provide the average spatial pattern of disease risk over the analyzed period, so cannot reflect the time pattern of disease risk [2]. However, quantifying the distributions of disease risk in space and time is important for understanding spatio-temporal phenomena (e.g. disease occurrence and its dynamics) [1]. Uncovering the full spatio-temporal profiles of disease risks may considerably strengthen the epidemiologic interpretations in the following three aspects [2]: depicting the changes of overall disease risks; pinpointing the stable high-risk regions throughout the whole period where potential risk factors are relatively fixed; and identifying unstable high-risk regions over time where there is substantial variability for the patterns of disease risk, which may prompt that potential risk factors are more variable. These results are useful for decision-makers to develop control strategies for different types of risk regions.

Kernel density estimation (KDE) provides a simple and flexible way of modeling data and has become an important nonparametric approach toward estimating distribution pattern of disease risk [3], [38], [39]. There is an important limitation, however, that the traditional KDE can only deal with continuous data. This study proposed a new idea of using so-called generalized product kernels to construct the stKDE to deal with spatio-temporal data of mixed data types. Through Monte Carlo simulations, we demonstrate that the proposed stKDE has a smaller MISE and narrower variation compared with trKDE under all the combinations considered. Appreciable improvements in finite-sample performance increase as the number of time periods increases. By analyzing two epidemiological examples using stKDE-based RRF, we further demonstrate that the efficacy of the proposed approach is satisfactory because stKDE not only makes use of the full sample rather than resort to sample splitting [18] but also borrow information from neighboring points in spaces and adjacent times periods, thus producing more stable estimation [14]. For the Burkitt's lymphoma data, Bailey and Gatrell used the *k*-function based approach to analyze this data set and concluded that significant space-time clustering existed [32]. Our proposed approach is from local cluster's perspective and it can further provide visual summaries on disease high-risk regions, its significance and dynamic changes. Since there were no controls available for the raw dataset, the results relied on the representativeness of selected controls. The purpose of this example was to show that our method can also be used jointly with sampling techniques which is important for epidemiological studies. For the data of schistosomiasis, the selected controls were reasonably representatives of the population at risk. In addition to the previous analyses [3], [35], the time information was included in the present analysis. Spatio-temporal pattern of disease risk will help to further characterize the spatial high risk regions that are stable or not over time [2].

Our proposed stKDE has three advantages. Firstly, as a nonparametric method, it places modest assumptions on the data structure. Secondly, it can handle the case involving mixed data types which is not possible for trKDE. Also, the basic idea of generalized product kernels can be easily extended to other more complicated situations of mixed data types such as mixtures of continuous, ordered and unordered categorical variables. Finally, it is simple to construct the stRRF for evaluating disease risk under different conditions and easily extended to other new functions such as excess risk function. Corresponding to the widely used parametric methods of Bernoulli and Poisson spatial-temporal scan statistic and spatio-temporal permutation scan statistic [40], the method of stRRF presented in this study can be extended to construct the weighted stRRF with counts and population at risk as weights and stKDE-based spatio-temporal permutation analysis, respectively. There are various possibilities for improving and extending the method described here and these are the subjects of continuing research. However, the previously popular scan statistic can only provide the information on when and where there are clusters, whereas other useful information is neglected such as the dynamic changes of overall risk and clusters which can be displayed as stated above.

There are three possible disadvantages for the stKDE method. Firstly, it is computationally intensive. To deal with this, parallel computing approaches have been developed to alleviate the computational burden associated with large dataset [18]. Secondly, multiple testing for the method of stKDE-based stRRF may result in false positive results. Thirdly, its statistical efficiency may be slightly lower than parametric method when the parametric models are correctly specified. As a result, false negative results may appear. However, true data generating process is always unknown beforehand [3], and this may be not that serious because the inverse effects caused by multiple testing may cancel it to some extent.

stKDE belongs to the technique of KDE, and therefore the problems of edge effect and bandwidth selection deserve a brief discussion. Edge effects are due to the fact that the information used to construct a map is spatially and temporally censored, i.e. the map and the study period has a border/limit and information from outside the study region and period is missing [13]. The current recommended solution for trKDE to this boundary problem is to apply explicit edge corrections, but it cannot be easily extended to higher dimensions with mixed data types [3], [7]. Thus, the generated density maps for individual cases and controls may suffer from the impacts of edge correction. However, we conjecture that the impact of the edge effect can be alleviated for stRRF to some degree because of possible cancellation by divisions between the case and control densities [3], [5], [41]. Bandwidth selection is another important topic. For stKDE, only the likelihood-based cross validation was explored to select the smoothing parameter, how about the other methods to choose optimum bandwidths? Are there important differences among different methods? For spatial RRF, the use of separate bandwidths has advantages in theories for 2 dimensional situations, particularly when case and control densities or their sample sizes are very different. However, in practice some authors argue that a common bandwidth is better than separate bandwidths, especially when the densities of cases and controls are equal [6], [29], [42]. Whether this applies for stRRF is not known, so separate bandwidths are applied in the present study. All these questions deserve further studies, which, unfortunately, cannot be solved in a short-term period because many feasible ideas/methods in spatial settings cannot be easily extended to spatio-temporal cases. The main aim of this study is to show the promising of the new idea on dealing with spatio-temporal studies in the field of epidemiology.

In summary, this study demonstrates a new idea of generalized product kernels and to demonstrate the promising of stKDE and stRRF for describing the patterns of spatio-temporal disease risks by including the time dimension. We believe that this is a competing method for spatio-temporal data analysis and would strongly encourage other researchers to explore this method further for better understanding of its theories and applications.

## Supporting Information

Text S1 **Brief introduction on the theoretical underpinnings of stKDE.** The theoretical underpinnings of stKDE are briefly introduced here and more references are pointed out for interested readers.
(PDF)

Figure S1 **trKDE Spatio-temporal relative risk surface showing the risk changes of Burkitt's lymphoma in the Western Nile district of Uganda from 1961–1975.** The degree of risk is denoted by the shade of gray with black shading representing the highest risk and white the least risk. The solid contour lines delineate the significant high risk regions. Compared to stKDE, trKDE is substantially less efficient and far less useful in small-sample settings as these examples clearly illustrate.
(TIF)

Figure S2 **trKDE Spatio-temporal relative risk surface depicting the dynamic changes of schistosomiasis risk in the Guichi region of China from 2001–2006.** The degree of risk is denoted by the shade of gray with black shading representing the highest risk and white the least risk. The solid contour lines delineate the significant high risk regions. Compared to stKDE, trKDE is substantially less efficient and far less useful in small-sample settings as these examples clearly illustrate.
(TIF)
