# Supplementary material for: Nonparametric Evaluation of Dynamic Disease Risk: A Spatio-Temporal Kernel Approach
Source: PLoS One. 2011 Mar 15;6(3):e17381. doi: 10.1371/journal.pone.0017381 (PMC3057986; doi:10.1371/journal.pone.0017381)
Supplement: Text S1 — Brief introduction on the theoretical underpinnings of stKDE. The theoretical underpinnings of stKDE are briefly introduced here and more references are pointed out for interested readers. (PDF) [file pone.0017381.s001.pdf]

Appendix A. Brief introduction on the theoretical underpinnings of stKDE

Say the observed individuals are recorded by the realization $(s_{i,1}, s_{i,2}, t_i)$ where the triplet represents the latitude, longitude, and time $i = 1, \ldots, n$. We presume that the realizations of cases represent draws from an unknown distribution, that the data are stationary and follow a mixing process, and allow for unknown correlation among occurrences over space and time. In order to estimate $f(s_1, s_2, t)$ we consider a nonparametric density estimator of the form,

$$\hat{f}(s_1, s_2, t) = \frac{1}{n}\sum_{i=1}^{n} \frac{1}{h_1} k\left(\frac{s_{i,1} - s_1}{h_1}\right) \times \frac{1}{h_2} k\left(\frac{s_{i,2} - s_2}{h_2}\right) \times K_t(t_i, t; \lambda) \tag{1}$$

Equation (1) represents a nonparametric kernel estimator of an unknown density $f(s_1, s_2, t)$ where each observation represents the random occurrence of a case characterized by ordinates in space and time (stKDE). The (s_1, s_2) ordinates are numeric datatypes (i.e., real-valued) while the time variable is an ordered discrete datatype. Parametric estimation of densities consisting of a mix of datatypes is referred to as "parametrically awkward" [1, page 419]. The nonparametric method embodied in equation (1) is designed to effectively handle the mix of real-valued and discrete variables encountered here.

In equation (1), the function $k(\cdot)$ is a traditional kernel for real-valued datatypes (e.g. Epanechnikov or Gaussian, see [8] and [7]), $K_t(\cdot)$ is a kernel function appropriate for discrete datatypes such as that proposed by [1, page 419] for both unordered and ordered categories. The functions $k(\cdot)$ and $K(\cdot)$ are simply weight ('kernel') functions that are appropriate for estimating density/probability functions. The use of product kernel functions is commonplace in the literature (see [9, page 149-151]). [2] labelled product kernel functions appropriate for mixed datatypes 'generalised product kernels'. The bandwidths (h_1, h_2, λ) are chosen via cross-validation hence the estimate is completely data-driven. Though it may not be immediately obvious, the spatial-temporal setting that we consider here is a direct application of the method proposed in [3], hence a few words may be in order.

Though s_1 and s_2 are ordinates (latitude and longitude), the spatial nature of the data does not create problems in this setting. A density function $f(s_1, s_2)$ where s_1 and s_2 represent pairwise random draws from real-valued random variables can be consistently estimated using kernel methods under standard regularity assumptions. That is, if (s_1, s_2) represent (joint) draws from two real valued random variables, whether these measure, say, returns on two assets (draws from the real number line) or location in space (also draws from the real number line) makes no difference to the kernel estimator, provided that the distribution of disease in (s_1, s_2) space exists, is stationary, smooth, and has finite moments. If these assumptions hold then we can consistently estimate $f(s_1, s_2)$ using kernel methods without having to presume that the parametric distribution is known to the researcher. This case was treated in [8] and [7].

Nor does the discrete (ordered) nature of the time variable create problems in this setting. We convert calendar time to UNIX time (a system for describing points in time, defined as the number of days elapsed since midnight (UTC) January 1, 1970). If we let t denote random draws from a

discrete ordered random variable, it creates no problems for the kernel method for estimating $f(t)$ providing you use a kernel that is appropriate for discrete support processes, which we do. This case was treated in [1] and [5].

As noted above, the article [3] considers the case of nonparametric kernel density estimation defined over a mix of real-valued and discrete random variables such as $f(s_1, s_2, t)$, and the current setting involving (s_1, s_2, t) (latitude, longitude, time) defined in equation (1) is but one such applications of this approach. Note that [3] explicitly treats the real-valued and *unordered* discrete z case, while here we have *ordered* discrete z. However, the ordered case is addressed in [3] and the key point is that the theoretical results are unaffected by whether the discrete variable is nominal or ordinal.[1] Though [3] explicitly treats the iid case, it is straightforward to show that the kernel density estimator is consistent and that cross-validation delivers bandwidths that converge to the optimal bandwidths with probability one for stationary weakly dependent mixing processes. For details on how results for the iid case treated in [3] carry over to the weakly dependent setting, see Racine and Li (2007, pages 537-541, "18.1 Density Estimation with Dependent Data") which demonstrates how almost sure convergence rates and limit distributions remain *unchanged* for ρ or α-mixing processes. Identical results carry over for the mixed discrete-continuous case. In particular, note that the MSE convergence rate with weakly dependent data is the same as the independent case treated in [4, page 537, Theorem 18.1]. The presence of the discrete variable t does not alter these results, rather, it simply introduces a finite-sample bias term that is offset by the reduction in variance brought about by smoothing across the discrete covariates.

References

1. J. Aitchison and C. G. G. Aitken, *Multivariate binary discrimination by the kernel method*, Biometrika **63** (1976), no. 3, 413–420.
2. P. Hall, J. Racine, and Q. Li, *Cross-validation and the estimation of conditional probability densities*, Journal of the American Statistical Association **99** (2004), 1015–1026.
3. Q. Li and J. Racine, *Nonparametric estimation of distributions with categorical and continuous data*, Journal of Multivariate Analysis **86** (2003), 266–292.
4. ———, *Nonparametric econometrics: Theory and practice*, USA: Princeton University Press, 2007.
5. D. Ouyang, Q. Li, and J. S. Racine, *Cross-validation and the estimation of probability distributions with categorical data*, Journal of Nonparametric Statistics **18** (2006), no. 1, 69–100.
6. ———, *Nonparametric estimation of regression functions with discrete regressors*, Econometric Theory **25** (2009), 1–42.
7. E. Parzen, *On estimation of a probability density function and mode*, The Annals of Mathematical Statistics **33** (1962), 1065–1076.

[1] We have explicitly treated kernel estimation with unordered and ordered cases in [6]. There we note that "When some of the regressors are ordered discrete ones, we use the kernel function defined in equation(10), and modify the definition of $\mathbf{1}_s(\bar{v}, \bar{x})$ so that $\mathbf{1}_s(\bar{v}, \bar{x}) = \mathbf{1}(|\bar{v}_s - \bar{x}_s| = 1) \prod_{t=1, t\neq s}^{r_1} \mathbf{1}(\bar{v}_s = \bar{x}_s)$ when $\bar{x}_s$ is an ordered discrete variable. Then it can then be shown that the conclusions of Theorem 2.1, Theorem 2.2, Theorem 3.1 and Theorem 3.2 remain unchanged." Whether conducting kernel regression or kernel density estimation, it remains the case that the presence of ordered or unordered covariates leads to identical theoretical results.

8. M Rosenblatt, *Remarks on some nonparametric estimates of a density function*, The Annals of Mathematical Statistics **27** (1956), 832–837.
9. D. W. Scott, *Multivariate density estimation: Theory, practice, and visualization*, Wiley, New York, 1992.
