# Supplementary figures and images for: Nonparametric Evaluation of Dynamic Disease Risk: A Spatio-Temporal Kernel Approach
Source: PLoS One. 2011 Mar 15;6(3):e17381. doi: 10.1371/journal.pone.0017381 (PMC3057986; doi:10.1371/journal.pone.0017381)

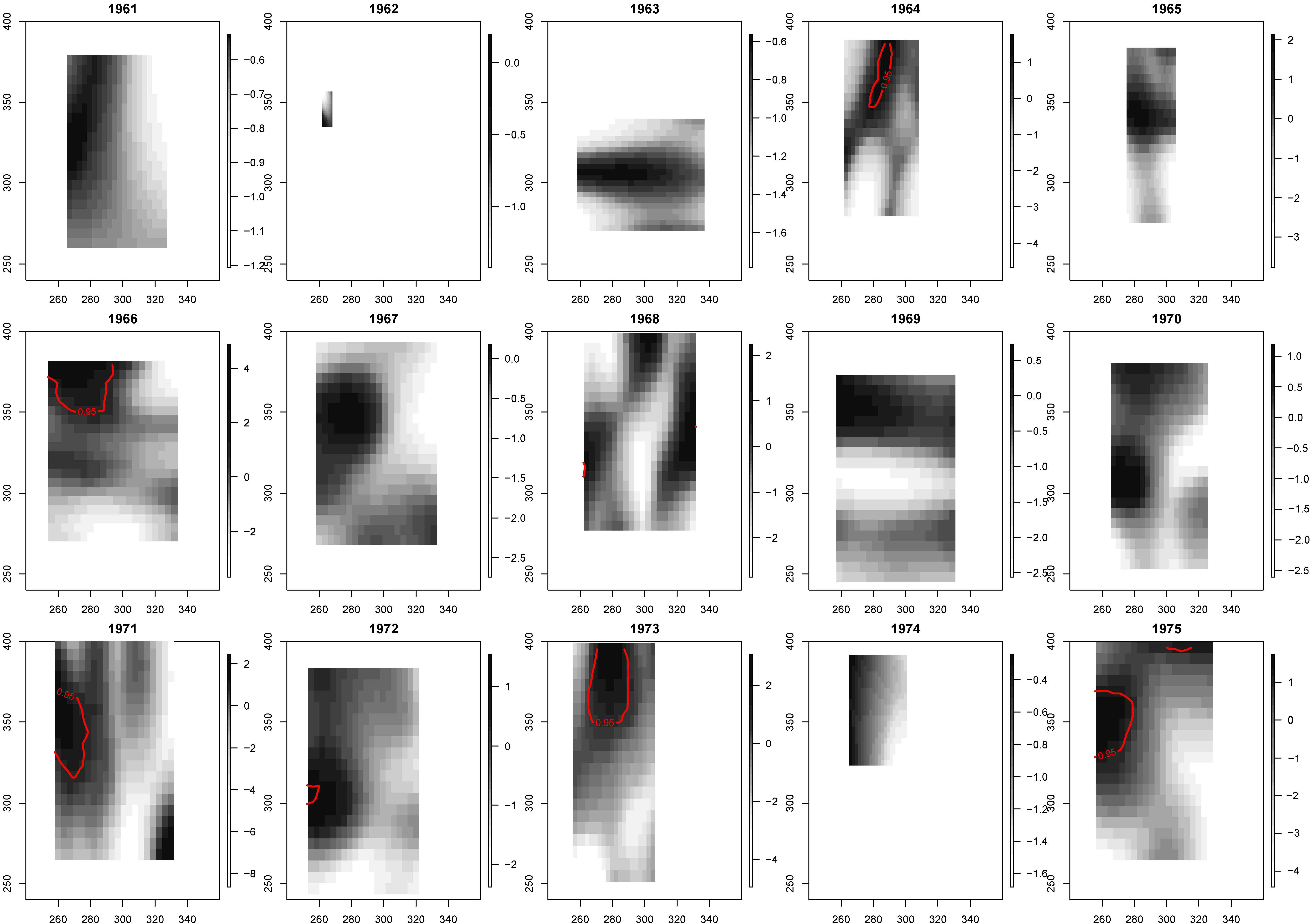

Supplement: Figure S1 — trKDE Spatio-temporal relative risk surface showing the risk changes of Burkitt's lymphoma in the Western Nile district of Uganda from 1961–1975. The degree of risk is denoted by the shade of gray with black shading representing the highest risk and white the least risk. The solid contour lines delineate the significant high risk regions. Compared to stKDE, trKDE is substantially less efficient and far less useful in small-sample settings as these examples clearly illustrate. (TIF) [file pone.0017381.s002.tif]

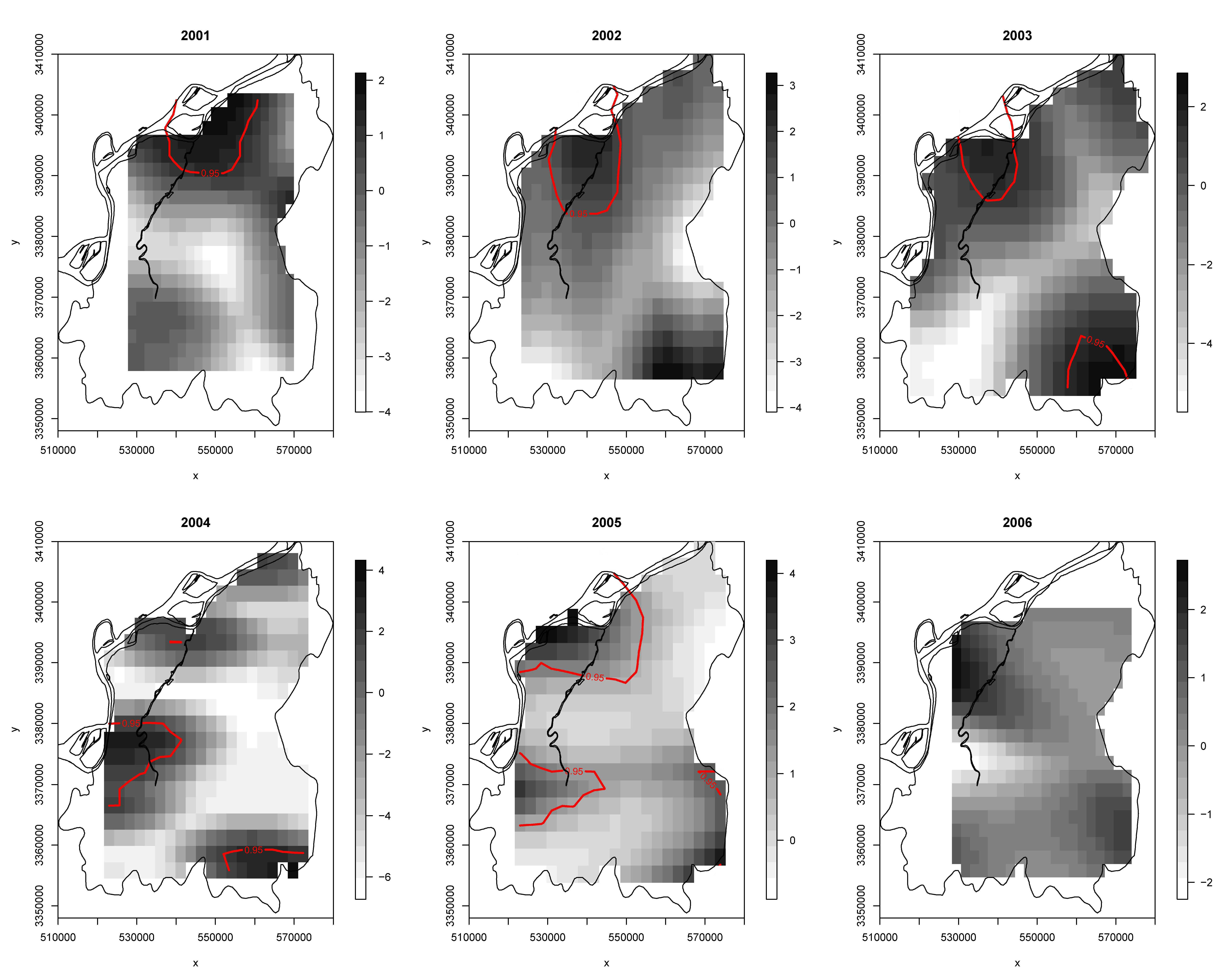

Supplement: Figure S2 — trKDE Spatio-temporal relative risk surface depicting the dynamic changes of schistosomiasis risk in the Guichi region of China from 2001–2006. The degree of risk is denoted by the shade of gray with black shading representing the highest risk and white the least risk. The solid contour lines delineate the significant high risk regions. Compared to stKDE, trKDE is substantially less efficient and far less useful in small-sample settings as these examples clearly illustrate. (TIF) [file pone.0017381.s003.tif]
